# Preoperative Considerations for Uterine Fibroid Removal in Patients With Mayer-Rokitansky-Küster-Hauser Syndrome and Klippel-Feil Syndrome: A Case Report

**DOI:** 10.7759/cureus.103907

**Published:** 2026-02-19

**Authors:** Yasuho Yanagihara, Yu Kawasaki, Keisuke Murakami, Taihei Yamada, Mari Kitade

**Affiliations:** 1 Obstetrics and Gynecology, Juntendo University Hospital, Tokyo, JPN

**Keywords:** klippel-feil syndrome, laparoscopic myomectomy, magnetic resonance angiography, mayer-rokitansky-küster-hauser syndrome, uterine fibroids

## Abstract

Mayer-Rokitansky-Küster-Hauser syndrome (MRKHS) is a congenital disorder characterized by dysplasia of the uterus and upper vagina due to Müllerian duct dysplasia. Fibroids arise from the rudimentary uterus. The implementation of surgical intervention for uterine fibroids in patients with MRKHS presents several challenges and considerations. The rudimentary uterus is tiny, making it difficult to determine the location of the pelvic mass and diagnose uterine fibroids. Preoperative planning is often inadequate, necessitating intraoperative decision-making. In addition, patients with MRKHS may present with Klippel-Feil syndrome (KFS), characterized by cervical spine fusion, further complicating intraoperative management. In contrast to the typical practice, careful surgical planning along with anesthesia administration and orthopedic surgery is required. In this case, magnetic resonance angiography was utilized to determine the location of the mass and the rudimentary uterus. Screening was conducted to detect any concomitant malformation, such as KFS. Subsequently, a safe laparoscopic surgery was carried out. In this case report, we present the importance and unique considerations of preoperative planning for the surgical intervention of uterine fibroids in patients with MRKHS.

## Introduction

Mayer-Rokitansky-Küster-Hauser syndrome (MRKHS) is a congenital disorder characterized by aplasia of the uterus and upper vagina caused by Müllerian duct aplasia. MRKHS is classified into Type 1 and Type 2. Type 1 is characterized by isolated uterovaginal aplasia, while Type 2 is marked by the coexistence of extragenital malformations and uterovaginal aplasia [1]. Müllerian duct aplasia may be associated with extragenital malformations involving the kidneys and skeleton [2].

Although the development of leiomyomas from rudimentary uterine tissue is theoretically possible due to the presence of smooth muscle cells in the proximal Müllerian ducts, it remains a rare occurrence, with only a limited number of cases reported in the literature [2,3].

The surgical management of uterine fibroids in symptomatic MRKHS patients presents unique diagnostic and therapeutic challenges. The rudimentary uterus is small, and identification of the ovaries poses a challenge, thereby complicating the clear determination of the mass's location and the formulation of a preoperative surgical strategy. A current study also faced challenges in preoperatively identifying ovarian tumors or uterine fibroids, often requiring an intraoperative confirmation for a definitive diagnosis [2]. Only a limited number of studies provide insights into the decisions made preoperatively and intraoperatively when selecting the surgical approach.

Furthermore, MRKHS Type 2 is frequently associated with skeletal malformations, occurring in approximately 28% of patients [4]. Among these, Klippel-Feil syndrome (KFS) presents particular concerns for anesthetic management due to restricted neck mobility, which increases the risk of neurological injury during tracheal intubation and necessitates specialized preoperative planning in collaboration with anesthesiology and orthopedic surgery.

This case report aims to highlight the critical importance of comprehensive preoperative evaluation in MRKHS patients with uterine fibroids, specifically emphasizing the utility of magnetic resonance angiography (MRA) for mass localization and the necessity of multidisciplinary collaboration when associated skeletal malformations are present.

The study was approved by the Juntendo University Research Ethics Committee. Informed consent was obtained from the patient.

## Case presentation

This was a case of a 36-year-old female patient. At the age of 20 years, she sought medical attention due to complaints of primary amenorrhea. She was diagnosed with MRKHS Type 2 with a vaginal defect, a rudimentary uterus, a normal female karyotype (46, XX), and normal gonadal function.

**Figure 1:**
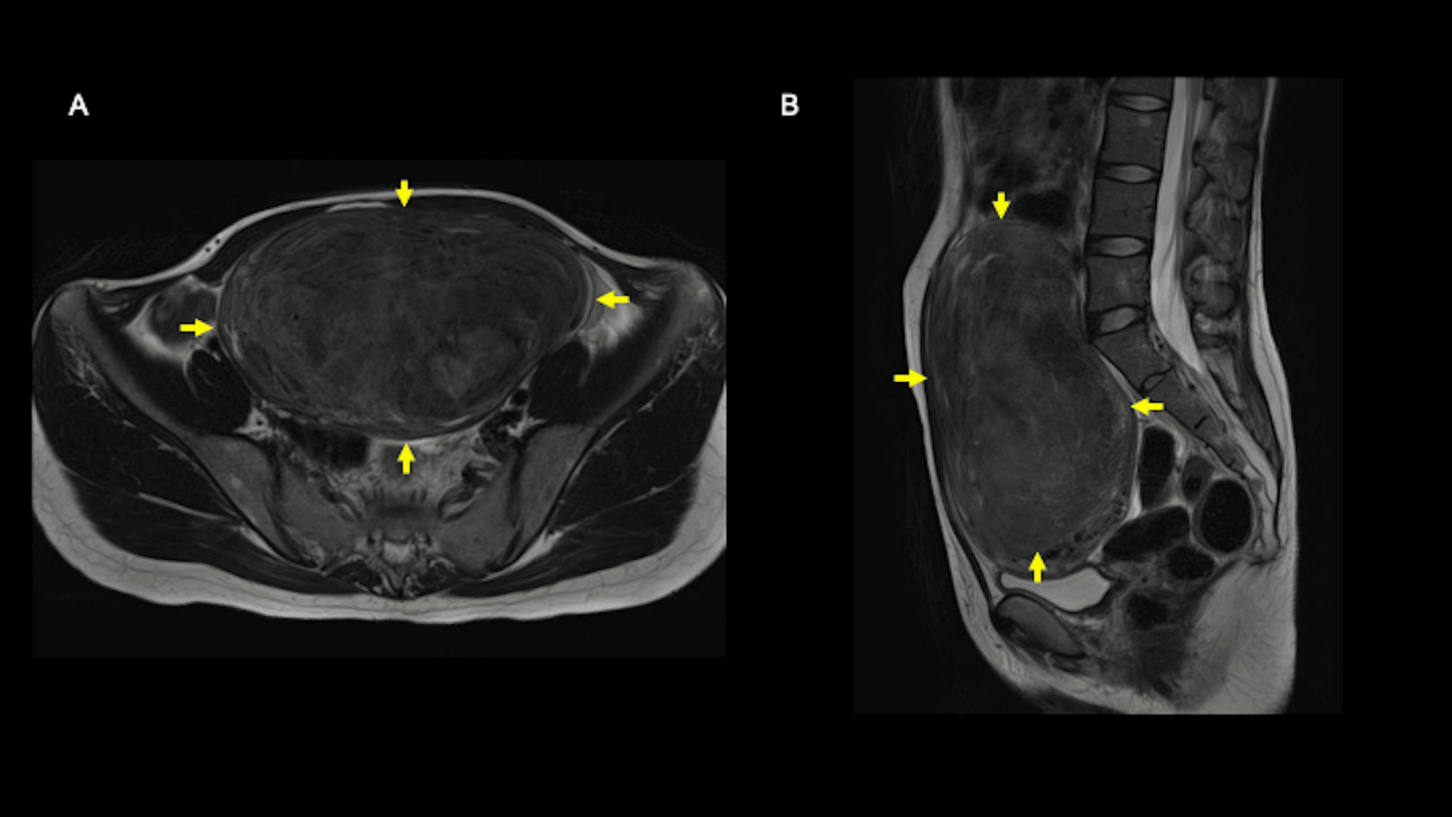
T2-weighted magnetic resonance imaging shown in the axial (A) and sagittal (B) views A 16-cm pelvic mass (yellow arrow) of unknown location in relation to the uterus and ovaries was detected.

The local doctor did not provide a detailed explanation of MRKHS, and no treatment, such as vaginoplasty, was recommended. At the age of 36 years, she presented to our hospital due to complaints of abdominal distension and a pelvic mass lesion measuring 16 cm in diameter. The patient experienced a substantial decline in her quality of life due to abdominal distension and was indicated for surgical intervention. Magnetic resonance imaging (MRI) revealed a myoma-like mass with a diameter of 16 cm adjacent to the left scarred uterus (Figure 1).

**Figure 2:**
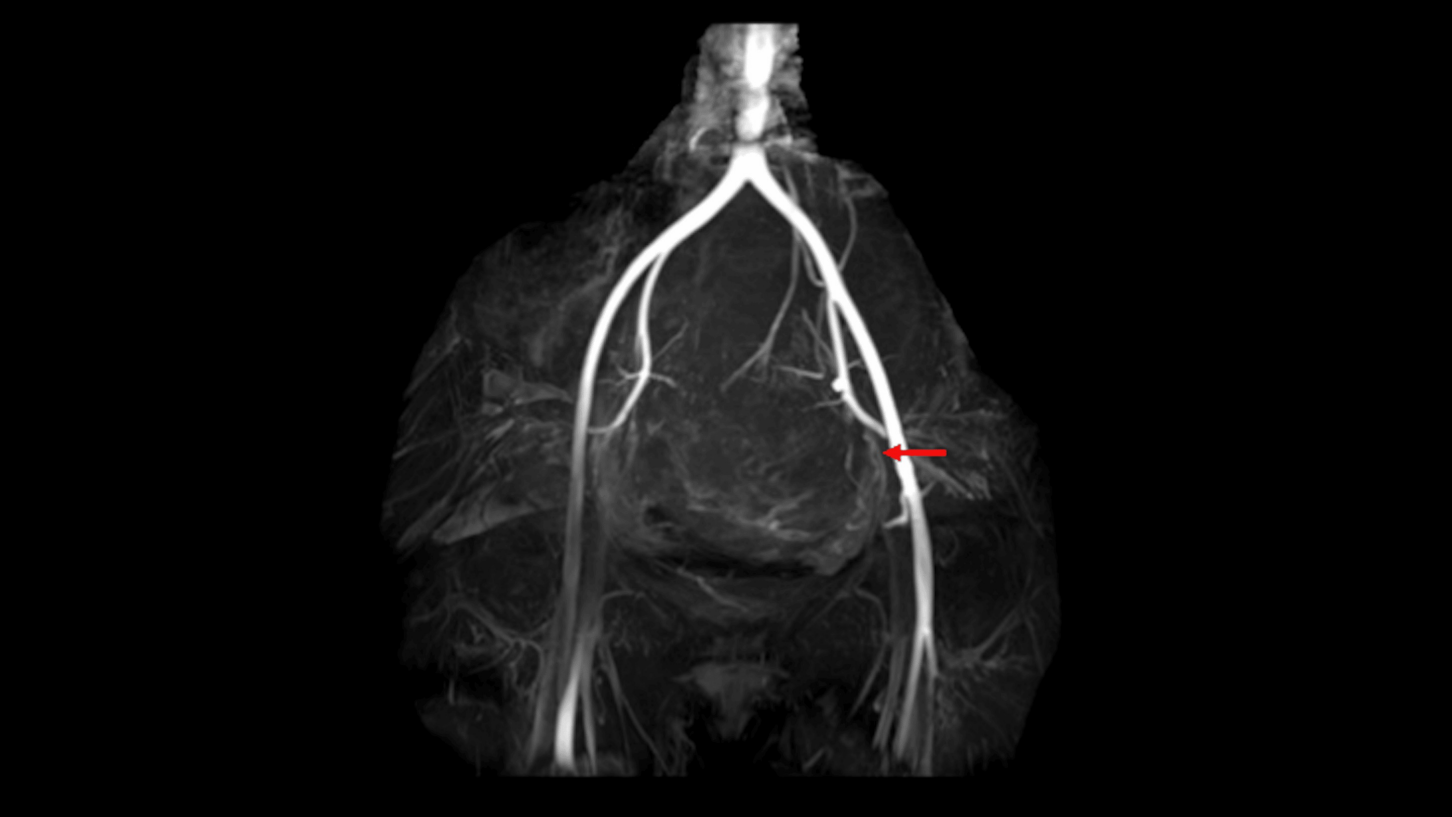
Pelvic magnetic resonance angiography showing the uterine artery (red arrow) branching from the left internal iliac artery to nourish the pelvic mass This finding was critical in establishing the uterine origin of the mass, distinguishing it from an ovarian tumor, and confirming the diagnosis of a uterine fibroid arising from the rudimentary uterus.

The precise localization remained uncertain, and distinguishing an ovarian tumor from a uterine fibroid was challenging. Magnetic resonance angiography (MRA) revealed an intra-abdominal mass with trophic vessels branching from the internal iliac artery, indicative of a uterine fibroid (Figure 2).

**Figure 3:**
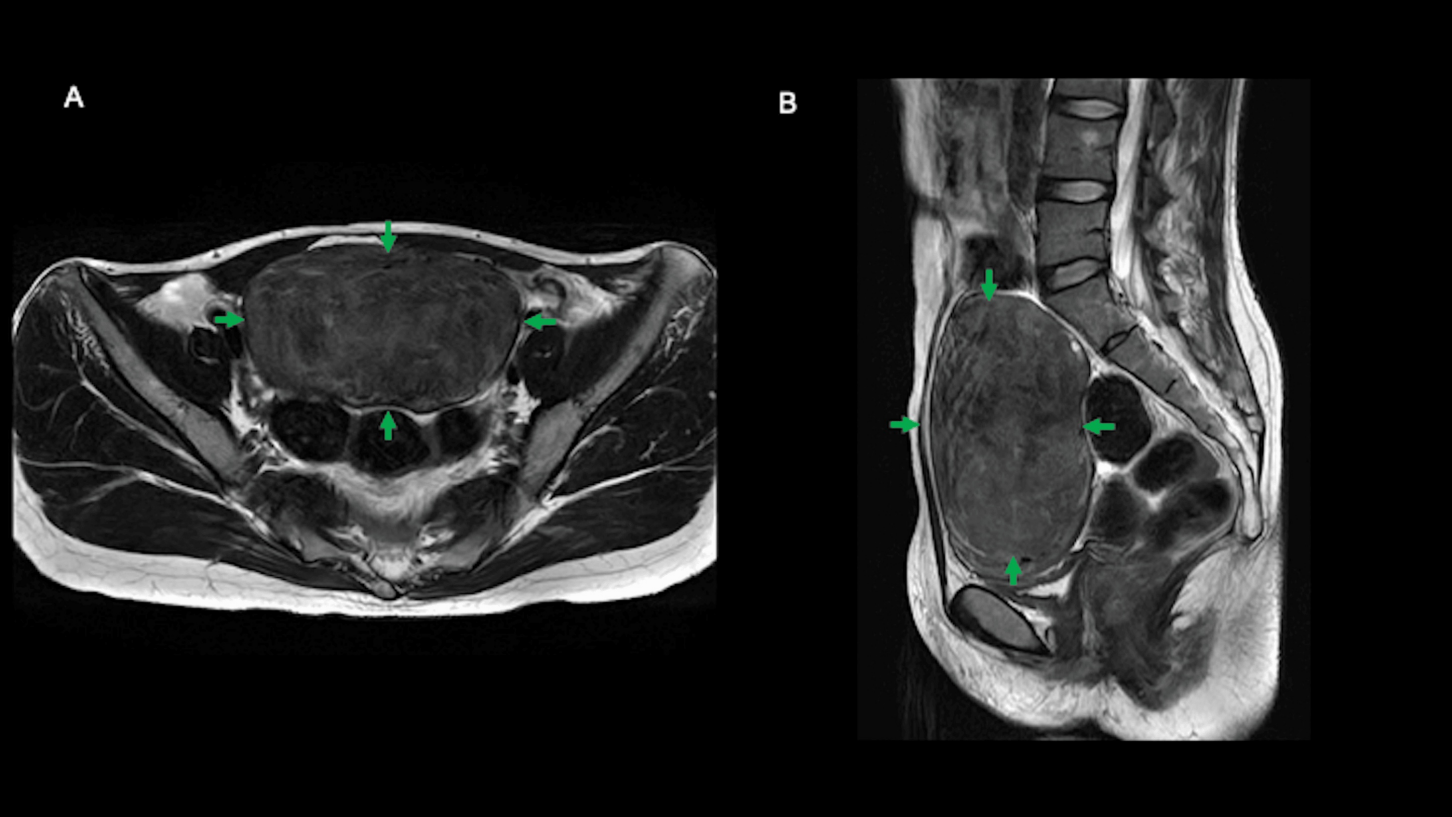
T2-weighted magnetic resonance imaging after preoperative hormone therapy with GnRH agonist (4 months), shown in the axial (A) and sagittal (B) views The pelvic mass (green arrow) showed a reduction in the maximum diameter to 12 cm.

Consequently, a laparoscopic total hysterectomy or laparoscopic myomectomy was planned. Preoperative hormone therapy with GnRH agonist was administered for 4 months, resulting in the reduction of the uterine fibroids to 12 cm (Figure 3).

**Figure 4:**
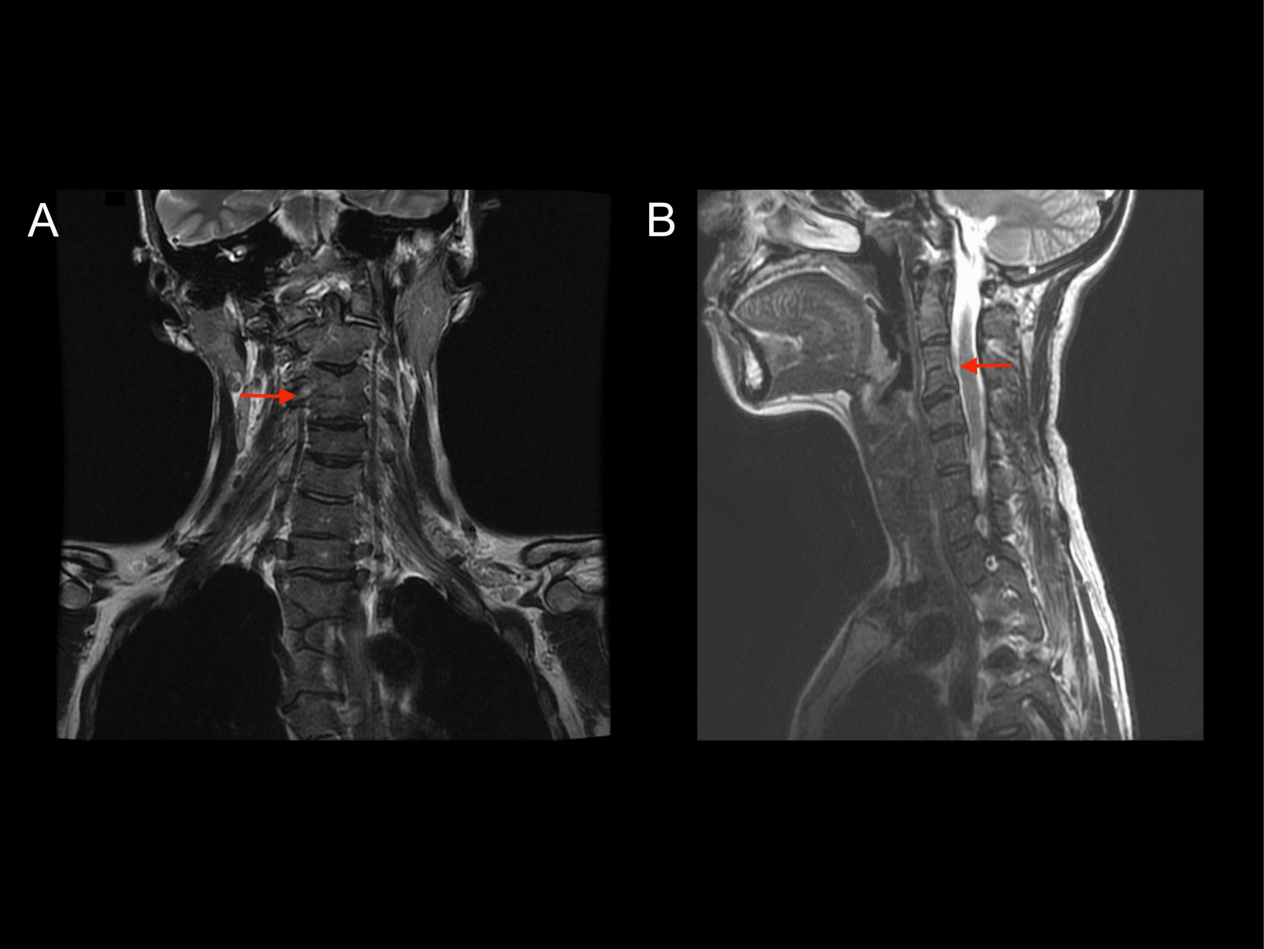
T2-weighted magnetic resonance imaging of the cervical spine and upper thoracic spine Cervical C3–4 fusion was noted (red arrow). The C4/C5 and C5/C6 discs are protruded.

 An additional examination for concomitant malformations revealed KFS characterized by cervical vertebral fusion (Figure 4).

Consequently, the orthopedic surgeon was consulted to examine the patient. The examination confirmed no limitation in forward flexion of the neck. However, limitations were observed in backward flexion (up to 20 degrees), right and left rotation (15 degrees), and right and left lateral flexion (20-30 degrees). She exhibited severely restricted cervical spine mobility, raising concerns about potential nerve damage during tracheal intubation with anesthesia. Therefore, a preoperative intubation simulation was performed in collaboration with the surgical and anesthesiology departments. 

Surgical procedure

**Figure 5:**
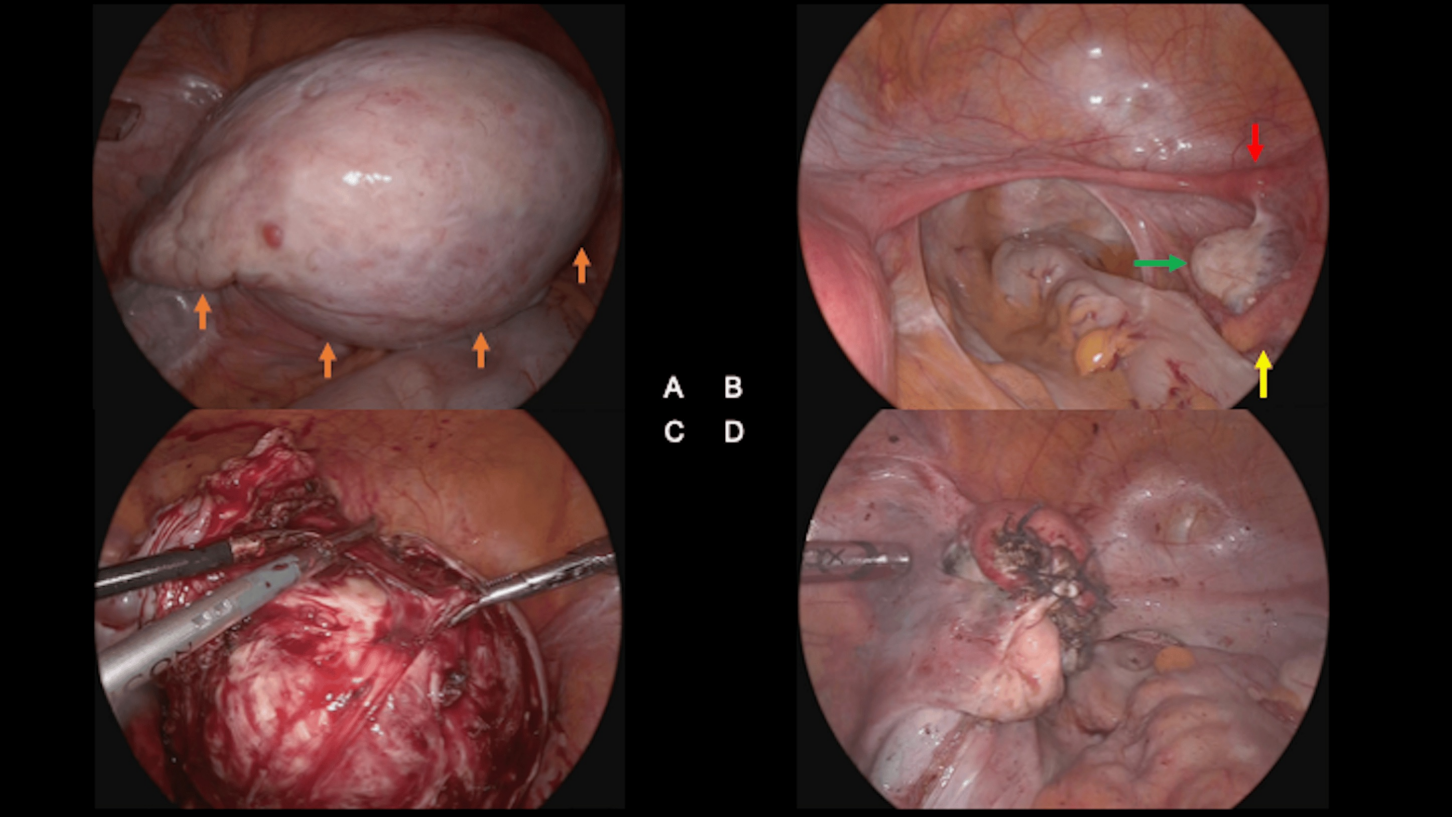
Intraoperative findings during laparoscopic myomectomy (A) The intra-pelvic mass (brown arrow) comprised the left rudimentary uterus, left ovary, and left fallopian tube as a single mass with indistinct borders. This further complicated the characteristic anatomical complexity and pelvic architecture distortion of MRKHS. Even with direct visualization, determining precise anatomical relationships remained challenging. (B) The pelvic floor, right rudimentary uterus (red arrow), right ovary (green arrow), and right fallopian tube (yellow arrow) were consistent with the MRKHS features, showing the typical MRKHS. (C) By incising the uterine muscle layer and enucleating the mass, it was possible to clarify its anatomical location and safely separate it from the ovary, confirming its uterine origin intraoperatively. (D) The first uterine muscle layer was closed using interrupted 1-0 absorbable thread sutures. Running sutures were used to close the second layer and serosa. Using that technique, we reconstructed the left rudimentary uterus.

Laparoscopic surgery was performed with the patient positioned in the lithotomy position with appropriate padding and leg support. The neck was maintained in an intermediate position for intraoperative management. A standard four-port laparoscopic technique was utilized. Intraoperative findings revealed a 12-cm mass with an indistinct border in the left ovary, connected to a cord-like object that appeared to be the left rudimentary uterus. The identification of the left fallopian tube proved challenging due to its unclear anatomy. Given the anatomical complexity, performing a laparoscopic total hysterectomy was deemed difficult. The decision was then made to proceed with a meticulous dissection and tumor removal, opting for a laparoscopic myomectomy (Figure 5).

The broad ligament of the uterus was unfolded, and the ureter was identified. Using MRA guidance for reference, the feeding vessels to the mass were identified and secured using an electrothermal vessel sealing system. The mass was then carefully dissected from the surrounding rudimentary uterine tissue. The specimen was retrieved by in-bag morcellation. The operative time was 155 minutes, and the estimated blood loss was 20ml. No intraoperative complications were encountered. 

Postoperative course and outcomes

**Figure 6:**
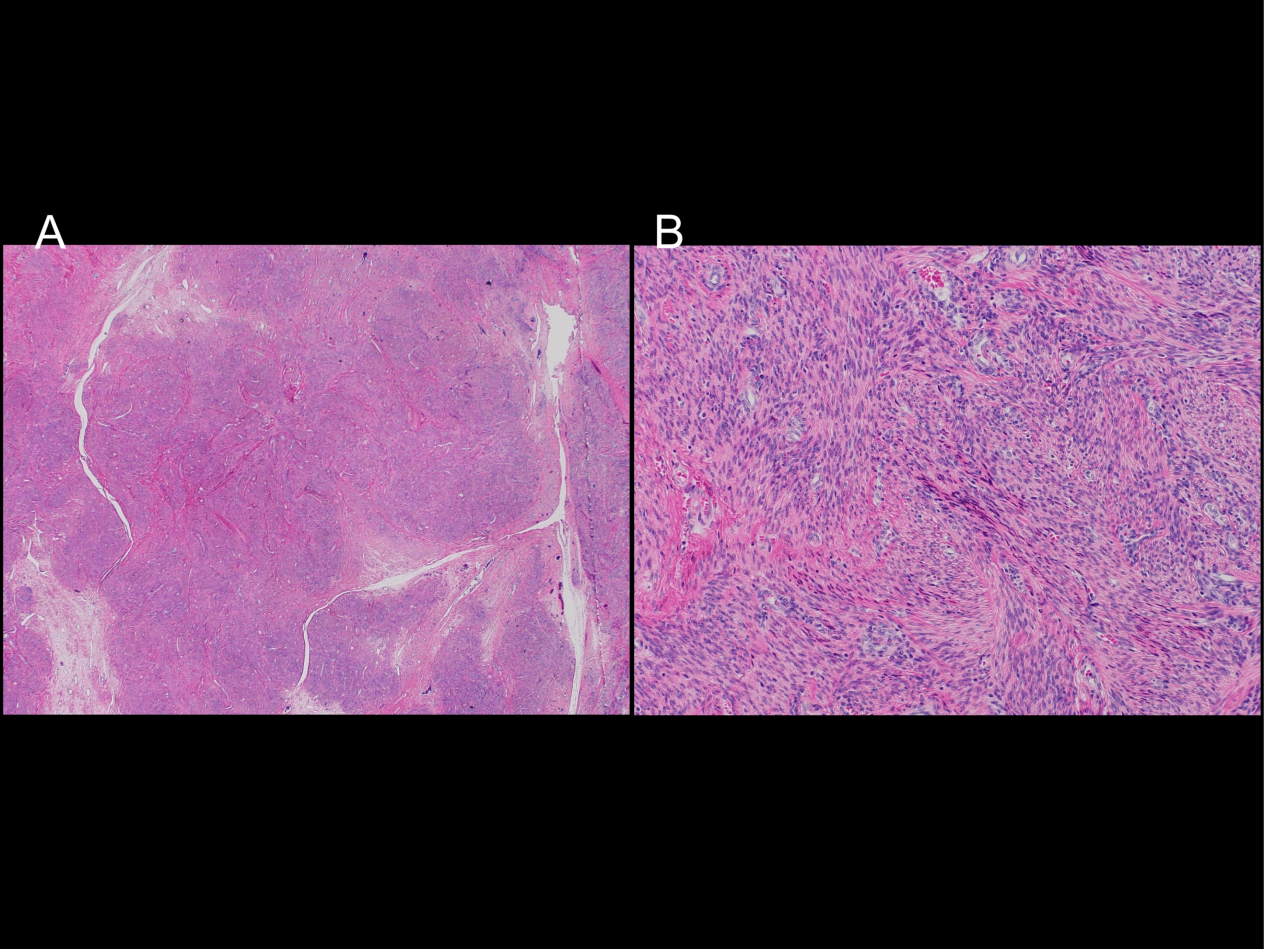
Pathological findings of the resected pelvic mass (left: HE×12.5, right: HE×100) The cells exhibited a complex, spindle-shaped proliferation, leading to the diagnosis of leiomyomas. HE: hematoxylin and eosin stain

The postoperative pathological examination confirmed that the tumor was a leiomyoma with spindle-shaped smooth muscle cell proliferation (Figure 6).

The postoperative course was favorable, and the patient was eventually discharged on the third postoperative day. The patient has been followed up for 24 months postoperatively, and no recurrence or symptoms have been reported to date.

## Discussion

Since 1977, several studies have reported the occurrence of uterine fibroids in patients with MRKHS [5]. This case demonstrates important considerations for the management of uterine fibroids in patients with MRKHS. Additionally, the combination of MRKHS and KFS presented unique challenges that required careful preoperative planning and multidisciplinary collaboration.

First, the utilization of MRA proved extremely valuable in this case for multiple reasons. Due to the altered pelvic anatomy, conventional MRI alone was insufficient to clearly distinguish between an ovarian tumor and a uterine fibroid. Furthermore, even with direct visualization of the abdominal cavity, determining the anatomical positional relationships between organs remained challenging. MRA has been reported as useful for evaluating uterine and ovarian arterial anatomy [6]. In this case, MRA clearly demonstrated the uterine arterial branch supplying the mass, definitively establishing its uterine origin. This distinction enabled a surgical procedure similar to myomectomy, involving incision through the myometrium for mass removal. While alternative diagnostic approaches, such as serum tumor markers or contrast-enhanced MRI, are sometimes used to differentiate uterine from ovarian masses, these modalities have important limitations in MRKHS patients. Tumor markers lack specificity, as CA-125 can be elevated in approximately 20-40% of patients with uterine fibroids, particularly those with large fibroids (≥5 cm) or coexisting adenomyosis [7-9]. Although contrast-enhanced MRI can provide vascular information, MRA offers distinct advantages as a minimally invasive technique that does not require contrast agent administration and provides more direct visualization of arterial blood supply. This approach was particularly useful in helping young women with MRKHS who already had reproductive issues to avoid complications associated with unnecessary oophorectomy and long-term hormonal effects, thereby facilitating the decision to undergo ovarian-sparing surgery. Previous case reports have described uterine fibroids arising from different locations in patients with MRKHS, including the retrovesical space, right broad ligament, right Müllerian bulb, right-sided intraperitoneal space of the round ligament, and right adnexal area [2]. These cases emphasize the importance of careful preoperative imaging and intraoperative decision-making in this patient population. Therefore, using minimally invasive MRA to confirm the relationship between the pelvic mass, uterine artery, and rudimentary uterus is essential for establishing effective preoperative planning.

Second, patients with MRKHS may have skeletal malformations as associated anomalies that can hinder the performance of laparoscopic surgery [4]. A specific skeletal malformation that makes intraoperative anesthetic management difficult is KFS, a congenital deformity characterized by cervical vertebral fusion [10]. The incidence of KFS in patients with MRKHS remains unknown. However, several case reports have documented patients with MRKHS complicated by KFS [11]. KFS is often asymptomatic and carries a high risk of misdiagnosis or being overlooked [12]. The hyperextension of the neck during intubation can cause severe nerve damage [13]. It can be easily diagnosed by identifying cervical fusion on plain radiographs (anterior-posterior, lateral, and open-mouth odontoid views) [12]. Systemic scrutiny of complications is essential to prevent unforeseen complications during surgical intervention in patients with MRKHS.

## Conclusions

This case demonstrates the importance of comprehensive preoperative evaluation in patients with MRKHS requiring surgical intervention for uterine fibroids. MRA provides valuable information for mass localization and surgical planning when conventional imaging is inconclusive. Additionally, systematic screening for associated malformations, particularly skeletal abnormalities, such as KFS, is essential for safe anesthetic management and surgical planning.

The successful management of this complex case required multidisciplinary collaboration between gynecologic surgery, orthopedics, and anesthesiology. This approach should be considered the standard of care for patients with MRKHS and associated comorbidities requiring surgical intervention.

## References

[1] Herlin MK, Petersen MB, Brännström M (2020). Mayer-Rokitansky-Küster-Hauser (MRKH) syndrome: a comprehensive update. Orphanet J Rare Dis.

[2] Romano F, Carlucci S, Stabile G (2021). The rare, unexpected condition of a twisted leiomyoma in Mayer-Rokitansky-Küster-Hauser (MRKH) syndrome: etiopathogenesis, diagnosis and management. Our experience and narrative review of the literature. Int J Environ Res Public Health.

[3] Kulkarni MM, Deshmukh SD, Hol K, Nene N (2015). A rare case of Mayer-Rokitansky-Kuster-Hauser syndrome with multiple leiomyomas in hypoplastic uterus. J Hum Reprod Sci.

[4] Oppelt P, Renner SP, Kellermann A (2006). Clinical aspects of Mayer-Rokitansky-Kuester-Hauser syndrome: recommendations for clinical diagnosis and staging. Hum Reprod.

[5] Beecham CT, Skiendzielewski J (1977). Myoma in association with Mayer-Rokitansky-Kuester syndrome. Am J Obstet Gynecol.

[6] Mori K, Saida T, Shibuya Y (2010). Assessment of uterine and ovarian arteries before uterine artery embolization: advantages conferred by unenhanced MR angiography. Radiology.

[7] Moss EL, Hollingworth J, Reynolds TM (2005). The role of CA125 in clinical practice. J Clin Pathol.

[8] Babacan A, Kizilaslan C, Gun I, Muhcu M, Mungen E, Atay V (2014). CA 125 and other tumor markers in uterine leiomyomas and their association with lesion characteristics. Int J Clin Exp Med.

[9] Bischof P, Galfetti MA, Seydoux J, von Hospenthal JU, Campana A (1992). Peripheral CA 125 levels in patients with uterine fibroids. Hum Reprod.

[10] Hensinger RN, Lang JE, MacEwen GD (1974). Klippel-Feil syndrome; a constellation of associated anomalies. J Bone Joint Surg Am.

[11] Strübbe EH, Lemmens JA, Thijn CJ, Willemsen WN, van Toor BS (1992). Spinal abnormalities and the atypical form of the Mayer-Rokitansky-Küster-Hauser syndrome. Skeletal Radiol.

[12] Litrenta J, Bi AS, Dryer JW (2021). Klippel-Feil Syndrome: pathogenesis, diagnosis, and management. J Am Acad Orthop Surg.

[13] Daum RE, Jones DJ (1988). Fibreoptic intubation in Klippel-Feil syndrome. Anaesthesia.

